# Supplementary figures and images for: Liver stage P. falciparum antigens highly targeted by CD4+ T cells in malaria-exposed Ugandan children
Source: PLoS Pathog. 2025 Feb 24;21(2):e1012943. doi: 10.1371/journal.ppat.1012943 (PMC11906071; doi:10.1371/journal.ppat.1012943)

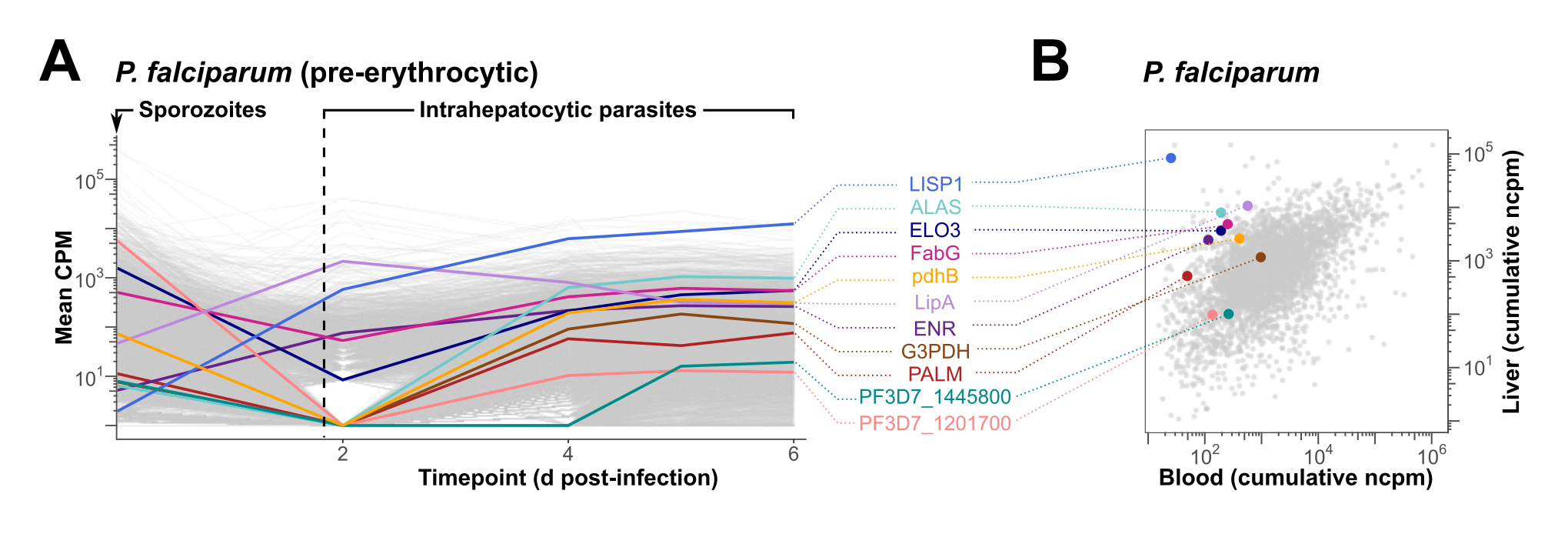

Supplement: S1 Fig — A. Gene expression in the pre-erythrocytic stages of P. falciparum (sporozoites obtained from Anopheles mosquito salivary glands and intrahepatocytic parasites obtained from humanized liver mice), as reported by Zanghi et al. [26]. B. Cumulative expression of genes in the blood and liver stages of P. falciparum derived from the datasets reported in Zanghi et al. [26] and Kucharski et al. [58]. Each point is a gene. Genes selected as candidate antigens are highlighted in color in A and B. (TIFF) [file ppat.1012943.s001.tiff]

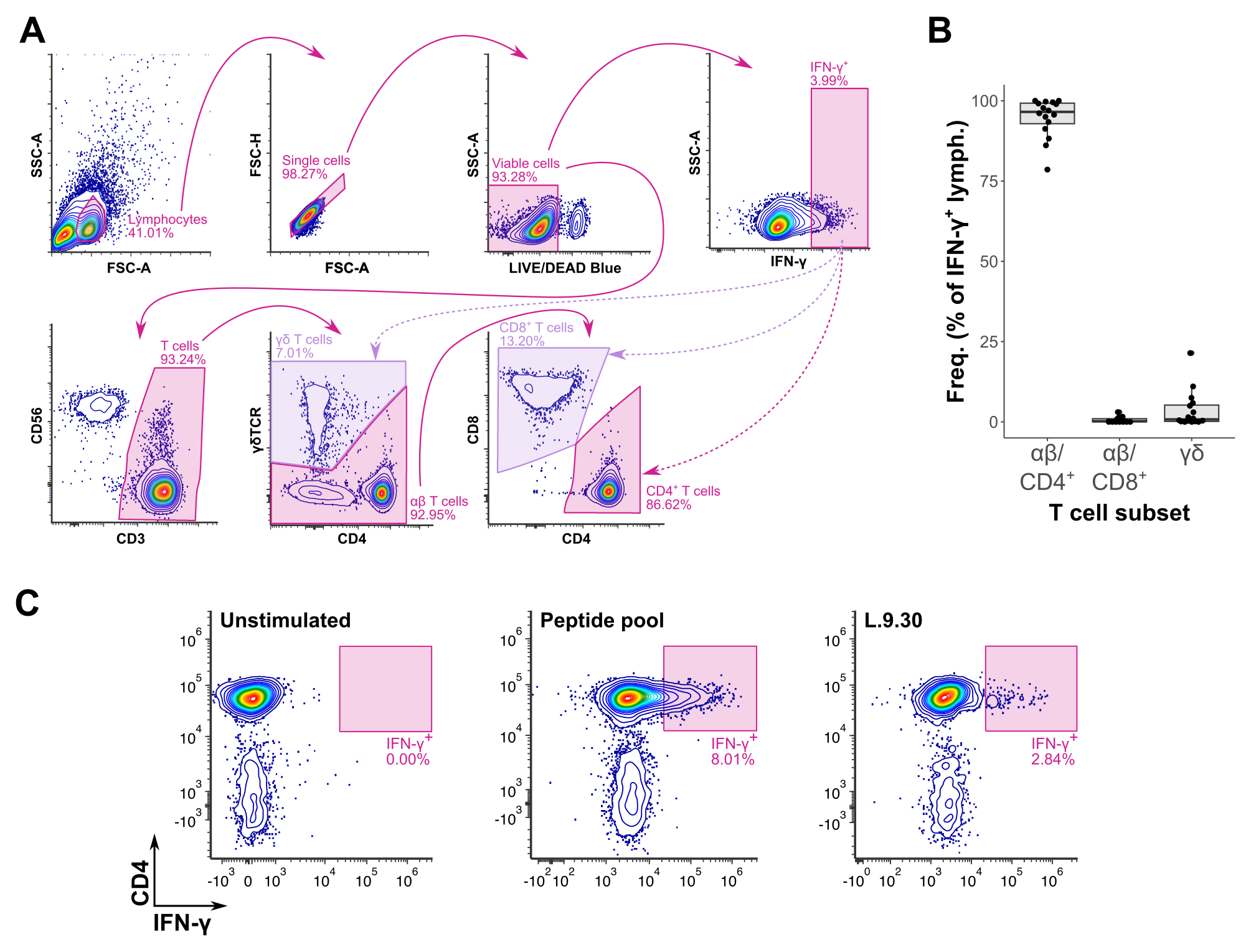

Supplement: S2 Fig — A. Gating strategy used to analyze flow cytometry data (one representative T cell line and stimulation condition shown). B. Distribution into T cell subsets of IFN-γ+ lymphocytes responding against stimulation with the total pool of candidate epitopes. Each data point represents a T cell line. Populations were defined by boolean combinations of the IFN-γ+ gate and the T cell subset gates as indicated by dashed arrows in A. C. IFN-γ production by CD4+ αβ T cells (gated upstream in lymphocytes/Single cells/Viable cells/CD3+CD56–/γδTCR–), after 18 h of culture in unstimulated condition or upon stimulation with the total pool of liver stage peptides, or peptide L.9.30 (cells from one representative donor are shown). (TIFF) [file ppat.1012943.s002.tiff]

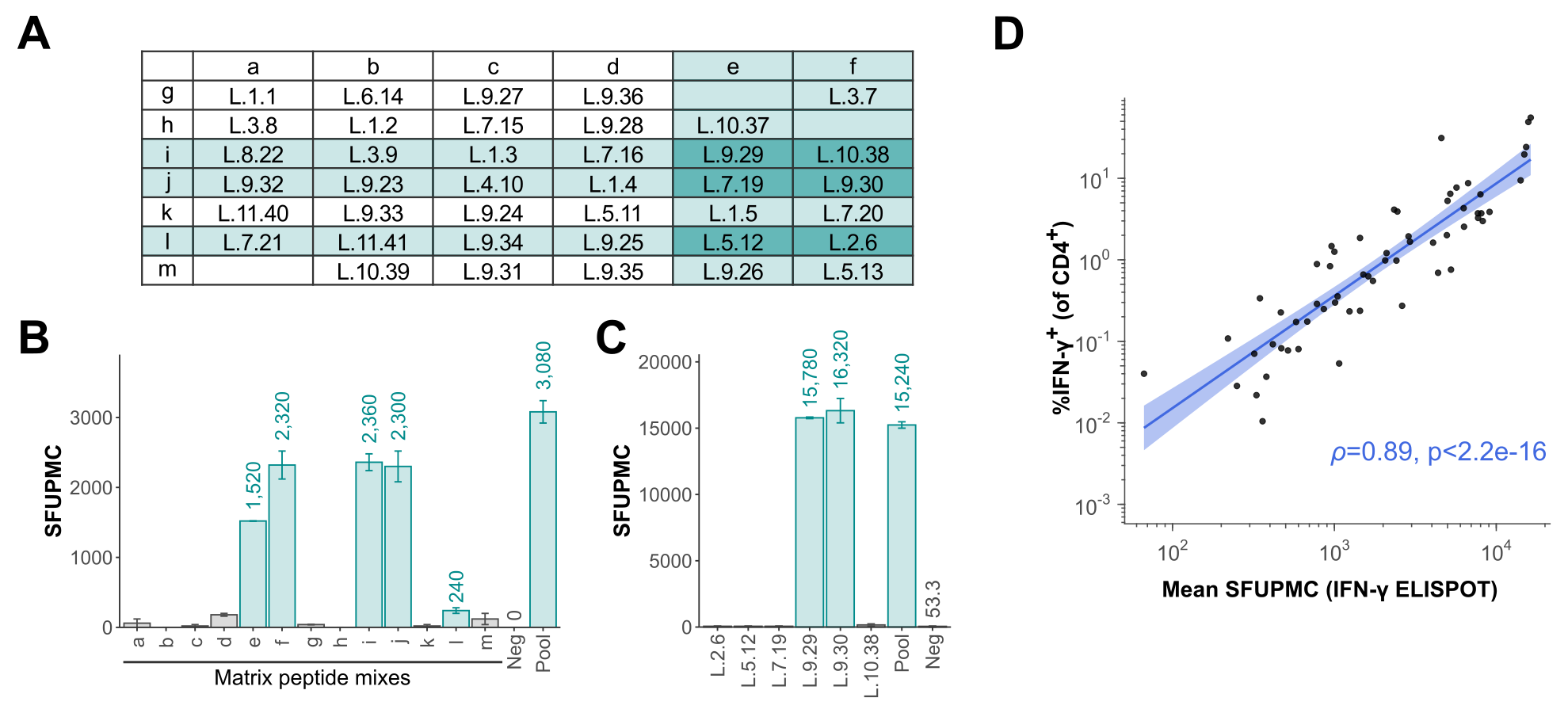

Supplement: S3 Fig — A. 2D peptide matrix used for first screening step by IFN-γ ELISPOT. A pool of peptides was created for each of the columns and each of the rows of the matrix. Short-term T cell lines were stimulated in IFN-γ ELISPOT experiments using each of these pools. B. Result obtained from one T cell line in the ELISPOT experiment described. The pools that elicited a response are highlighted in the matrix representation on panel A. C. Following the results from B, single peptides were identified at the intersections highlighted in A and tested individually in the second screening step (deconvolution). The resulting measured response pinpointed L.9.29 and L.9.30 as targets of the T cell line under study. D. Correlation between IFN-γ ELISPOT SFUPMC (day 17 of T cell culture) and frequency of IFN-γ+ events observed by flow cytometry (day 28 of T cell culture) for short-term T cell lines stimulated with individual peptides or the total pool of candidates. Line and error band represent adjusted robust linear regression. Spearman’s ρ and p-value are displayed. In B and C, numbers indicate the mean SFUPMC from duplicate-assayed wells for peptide-stimulated conditions, and triplicate negative control wells (Neg). Error bars show the maximum and minimum SFUPMC for each condition. SFUPMC: spot-forming units per 106 input cells. (TIFF) [file ppat.1012943.s003.tiff]

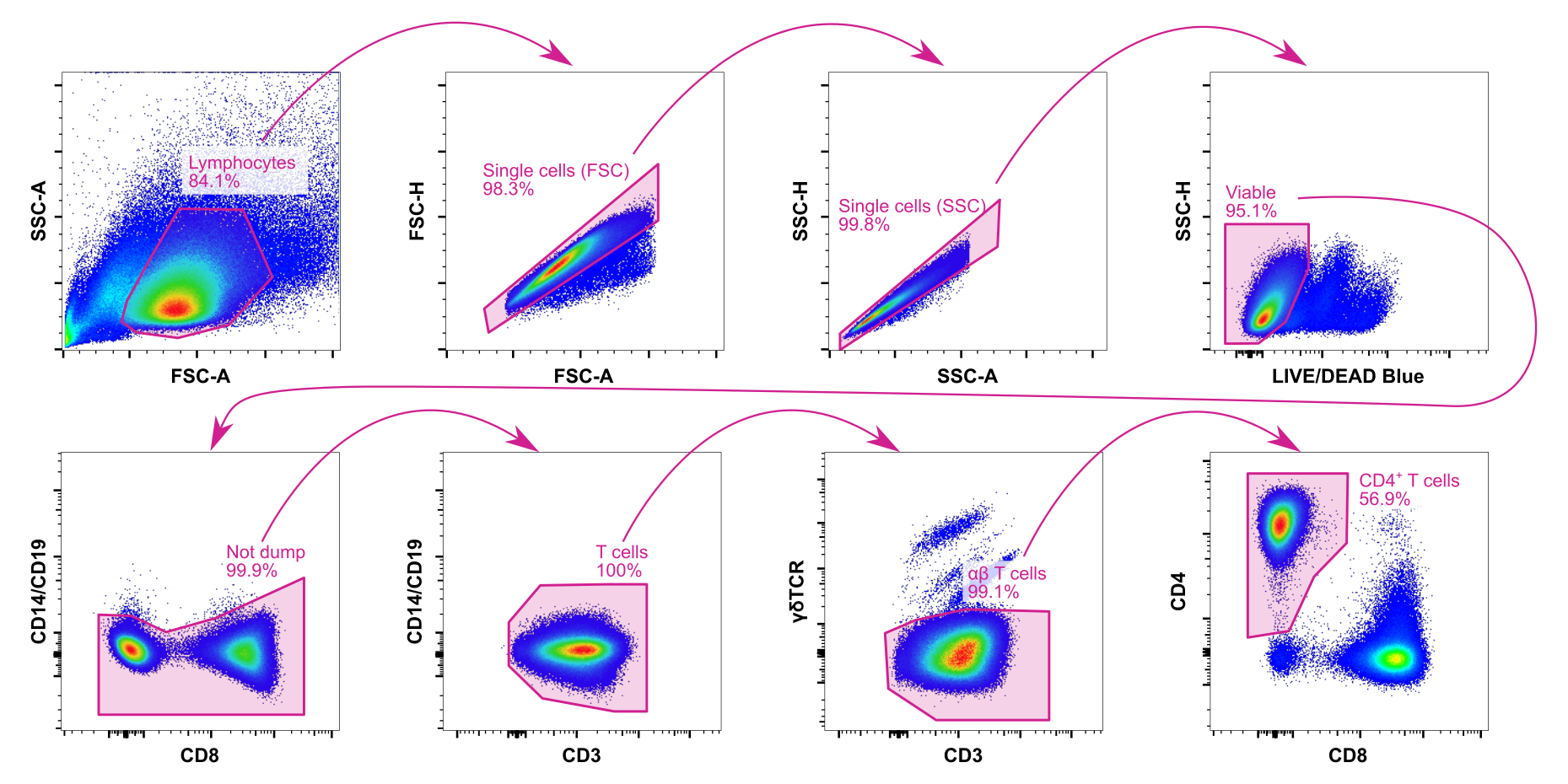

Supplement: S4 Fig — Flow cytometry gating strategy used to analyze pMHC multimer staining on primary T cell lines. Representative plots for one T cell line are shown. (TIFF) [file ppat.1012943.s004.tiff]
