# Supplementary material for: Liver stage P. falciparum antigens highly targeted by CD4+ T cells in malaria-exposed Ugandan children
Source: PLoS Pathog. 2025 Feb 24;21(2):e1012943. doi: 10.1371/journal.ppat.1012943 (PMC11906071; doi:10.1371/journal.ppat.1012943)
Supplement: Attachment — Submitted filename: Rebuttal letter_Final.pdf [file ppat.1012943.s005.pdf]

UCSF
University of California
San Francisco

Margaret Feeney, M.D.
Professor
Department of Medicine
Division of Experimental Medicine

UCSF at Zuckerberg
San Francisco General
Hospital and Trauma Center
Box 1234
1001 Potrero Avenue
San Francisco, CA 94110

tel: 628.206.8216
fax: 628.202.8091
Margaret.Feeney@ucsf.edu

www.ucsf.edu

December 8, 2024

Dr. Tracey J. Lamb, Section Editor
Dr. Michael Malim, Editor-in-Chief
PLOS Pathogens

RE: Revised submission for PPATHOGENS-D-24-02133 - [EMID:b7eda0947ff4ea45]

Dear Editors,

We would like to thank you for the opportunity to submit our revised manuscript, "Liver stage *P. falciparum* antigens highly targeted by $CD4^+$ T cells in malaria-exposed Ugandan children", to be considered for publication in PLOS Pathogens. We would also like to thank the Reviewers for their insightful comments and suggestions, which have improved the clarity and interpretability of our report.

Our point-by-point responses to the Reviewers' comments are highlighted in blue text on the following pages. All line numbers refer to the marked-up version of the revised manuscript. Numbering was added where absent to Reviewers' questions in order to facilitate cross-referencing between responses.

Thank you for your consideration.
Sincerely,

Margaret a Feeney

Margaret E. Feeney, M.D., MSc.
Edward B. Shaw Chair in Pediatrics
Professor, Division of Pediatric Infectious Diseases and Global Health

THE UNIVERSITY OF CALIFORNIA · LET THERE BE LIGHT · 1868

Part II – Major Issues: Key Experiments Required for Acceptance

Reviewer #1:

1. One of my main concerns, and a limitation of the study, is the assumption that the identified antigens are targets of protective T cell immunity. For example, in the abstract, it is stated that "those novel liver-stage P. falciparum epitopes represent promising new vaccine targets." However, the fact that T cell responses were detected in children from a highly exposed area with a recent clinical episode suggests that these responses are not protective and more likely reflect recent exposure. This point has not been discussed in the manuscript. Is there any data on subsequent malaria episodes or infections in these children? If so, correlates of protection analyses could be performed, although the sample size is limited. Alternatively, could samples from children without malaria (i.e., protected individuals) be analyzed?

We thank the reviewer for this comment. We agree that, based on the data included in this manuscript, we cannot claim that the described epitopes play a role in immune protection, and we have changed this sentence in the abstract to ensure that this is not implied (lines 25-27). We have also expanded the final paragraph of the manuscript to discuss the next logical steps needed to determine the role of these epitope-specific T cells in protection and the potential relevance of liver-stage antigens as vaccine candidates (lines 283-288).

We agree with the Reviewer that the next logical step is to determine whether T cell recognition of LISP1 (and the other liver-stage epitopes) is associated with prospective protection from malaria in larger cohorts; if so, these would represent attractive vaccine targets. Given the very small number of subjects included in the current study, we are underpowered to perform a robust analysis of protection. We have performed many such studies of correlates of T cell protection in the past, and typically include hundreds of subjects (e.g. PMID: 29097996) which enables us to adjust for known covariates of infection. Further, we selected PBMC samples for study based on having had a recent infection, and this introduces a bias that would be inappropriate for a proper cohort study of prospective protection. Nonetheless, as we do have clinical data on subsequent malaria outcomes, we performed the following analyses requested by the reviewer:

1) Mann-Whitney test comparing the time to next malaria episode between LS pool responders and non-responders ($p = 0.84$);
2) Spearman's correlation between the quantitative response (ELISPOT SFUPMC) to the LS pool and time to next malaria episode ($R = –0.086$, $p = 0.67$ for all donors; $R = –0.12$, $p = 0.23$ for LS pool responders only);
3) Mann-Whitney test comparing the time to next malaria episode between donors who responded to any LISP1 epitope and those who did not ($p = 0.42$);
4) Spearman's correlation between total response against LISP1 peptides and time to next malaria episode ($R = –0.04$, $p = 0.83$); and
5) Spearman's correlation between the number of distinct antigens targeted by each individual and time to next malaria episode ($R = –0.032$, $p = 0.86$).

Although we found no statistically significant association, given the very small number of study participants and the caveats above, we are likely underpowered and the risk of Type II error is high, so we feel it may mislead the readers to report that responses *are not associated* with prospective protection. Rather, we feel that the present manuscript reports the initial steps (identification of dominant epitopes and development of pMHC multimer tools) that will facilitate addressing important questions of protective immunity in larger cohorts of children.

2. Not clear if all peptides were tested in non-exposed negative controls for cross-reactive responses.

Yes, we tested PBMC from non-exposed control subjects for recognition of all peptides and found no evidence of cross-reactivity. We are grateful to the reviewer for pointing out our failure to include these details, which have now been added to the Results (lines 140-141 in the revised manuscript) and Methods (lines 436-439) sections. The total pool of liver stage candidate peptides was used to expand and test $CD4^+$ T cells from three malaria-naïve donors using the same protocol described for the Ugandan children cohort, detailed in Fig. 3A. No responses against the peptides were detected.

3. In Figure 4, donor 3059, the gating of IFN-g does not look ok for CLIP and L.9.30, the gate seems to cross the IFN-g negative populations (both the tetramer negative and positive).

We thank the reviewer for this observation. The gates were adjusted accordingly.

Reviewer #2: Major concern
1. Given the high response frequency observed here after in vitro amplification but lack of detectable response direct ex vivo, this reviewer would like to see a negative control where PBMC from malaria naïve donors are in vitro amplified-specifically with the LISP1 epitope pools and tested in the ELISPOT

Please see the response to point 2 raised by Reviewer #1.

Reviewer #3:
1. Please define Plasmodium-specific antibody titers in these children, especially the titers for LISP1-specific antibodies. This outcome measure could provide context for the amount of "help" the LISP1-specific T cells are providing to the LISP1-specific B cells.

This data would be difficult to generate, as the LISP1 protein is not commercially available nor are ready-made ELISA products to measure LISP1 antibodies. Optimizing a system for in vitro protein expression would require significant effort and time (LISP1 is a very large protein, likely requiring specialized expression systems – e.g. fragments of LISP1 have been successfully produced using the wheat germ expression system). Importantly, given its localization to the PV in intra-hepatocytic parasites, we would expect that LISP1 is unlikely to be exposed to circulation. Because of this, we hypothesize that anti-LISP1 antibodies do not substantially contribute to immunity against liver-stage *P. falciparum*. We think the most likely role of LISP1-specific $CD4^+$ T cells is either to modulate other effector lymphocytes (i.e. provide "help" to $CD8^+$ T cells) or to mediate cytotoxic function. Cytotoxic $CD4^+$ T cells have been implicated in immunity against *P. falciparum* (Furtado et al., 2023, Nature Communications), raising the possibility that LISP1-specific $CD4^+$ T cells could mediate direct killing of infected hepatocytes. Assessing functional profiles of liver stage-specific $CD4^+$ T cells (i.e. cytotoxic activity, Th1/2/17 cytokine secretion) is of great interest, and we are directing current efforts toward establishing protocols to enrich and phenotype these cells ex vivo, without the aid of in vitro expansion. Given all of these considerations, we feel that the measurement of anti-LISP1 antibody titers is outside the scope of the present manuscript.

2. To establish clinical significance of the epitopes defined in this report, it would be of interest to know follow up information about these children in terms of future malaria episodes in a given 3-12 month time period. It is tempting to hypothesize that children with more Plasmodium-specific CD4 T cells would have fewer malaria episodes. While correlation does not mean causation, this would be of interest if the information is available for the cohort of children.

Please see response to point 1 raised by Reviewer #1 above.

Part III – Minor Issues: Editorial and Data Presentation Modifications
Please use this section for editorial suggestions as well as relatively minor modifications of existing data

that would enhance clarity.

Reviewer #1:

1. The statement that the efficacy of RTS,S is lower in populations with greater malaria exposure is inaccurate. The reference provided also makes this assumption without enough evidence. In fact, there is no clear evidence that higher exposure is associated with lower efficacy or protection, with some studies showing a trend or a small effect, while others show no effect or even a correlation between higher CSP or malaria antibodies at baseline with greater immunogenicity, which is linked to higher protection. The endpoints in clinical trials are often confounded by factors related to malaria incidence, making it difficult to isolate the effects of transmission intensity. This statement should be revised or removed.

We have removed this sentence (and associated reference) from the introduction paragraph as suggested by the reviewer.

2. It is not clear how the 41 peptides were shortlisted from the 295 predicted peptides.

We expanded the Methods section of the manuscript to clarify the details of our selection process (lines 394-399 and 405-407).

3. Why were children selected for the study instead of adults, who would have had longer exposure and potentially higher immunity?

We chose to assess T cell responses in children because we have access to PBMC samples previously obtained from a large longitudinal cohort study (PRISM) that enrolled children between 6 mos and 11 years of age, and all children in this cohort had been HLA-typed for an earlier study, allowing us to select participants expressing our targeted DRB1 alleles. (Some adult caregivers were also enrolled PRISM, but they were far fewer in number than children, and fewer adult PBMC samples remain). We chose children 5-11 years old, as our previous studies indicate that Tororo children in this age range have already experienced substantial exposure to malaria and have acquired some degree of adaptive immunity. In addition, we selected samples that were obtained within 90 days of a clinical malaria episode (as we hypothesized that frequencies of Pf-specific cells would be higher after a recent infection), and this restriction would exclude most adult samples.

4. Some responders in the EliSpot assay had no detectable responses in the ICS assay. However, for those with positive responses in both assays, the correlation was quite good. Why do the authors believe that the ICS assay showed less sensitivity in some cases?

ICS assays are generally considered to be less sensitive than cytokine ELISPOT (Barabas 2017 BMC Immunol; Tischer 2014, J Immunol Methods; Karlsson 2003 J Immunol Methods). In the case of our results, it should be noted that ICS assays were performed on day 28 since stimulation, i.e. 14 and 11 days after the matrix screening (Fig 3B) and deconvolution (Fig 4A) ELISPOT experiments, respectively. This additional time was necessary to expand the primary T cells to adequate numbers for the flow cytometry assessment. The generally strong correlation observed between the results from the two methods (Fig 3C and S3D) suggests that the proportion of peptide-specific cells is well preserved during this gap between experiments. However, slight changes are not entirely unexpected, and their effect is presumably most noticeable on T cell lines with lower response magnitudes.

5. Lastly, in line 152, there is an error with a date and time inserted.

We apologize for the error; it has now been corrected.

Reviewer #2:
Minor concerns
1. Please check carefully for typos in text and references

This has been done.

2. Some discussion is warranted as to how the authors would proceed to prioritize these antigens for vaccine assessment
3. Some discussion is warranted as to how the authors would assess the effectiveness of vaccination against one or more prioritized antigens

We thank the Reviewer for these related suggestions. We believe that more evidence is necessary in order to explore how these antigens could be prioritized for vaccine development. Specifically, the association of responses against these antigens (in naturally exposed or experimentally infected subjects) and prospective protection from malaria would be a useful parameter to orient this decision. As discussed in detail in response to major issue #1 raised by Reviewer #1, the study presented here lacks adequate statistical power to test these correlations. Lines 260-264 and 283-288 of the revised manuscript were included to reflect this.

4. It is not exactly clear what criteria were used to downselect to the 11 candidate antigens

The criteria for selection of candidate antigens were 1) selective liver stage expression in both *P. vivax* and *P. cynomolgi* 2) low expression during the intraerythrocytic development cycle for *P. falciparum* 3) strongly correlated expression at matched life cycle stages between *P. falciparum* and other *Plasmodium* species (this is detailed in lines 97-102 and schematized in Fig.1). Further detail about the scoring function that was used to select antigens based on these criteria is in the Methods section (lines 364-392). Additionally, we have expanded the description of our epitope down-selection approach in the Methods section of the revised manuscript, which also determined the selection of 11 antigens (lines 394-399; please see also the response to minor issue #2 raised by Reviewer #1.)

Reviewer #3: Is it possible to perform tetramer enrichment on PBMCs from individual or pooled patients to begin to identify surface phenotypes of these antigen-specific CD4 T cells? HLA typing and blood volumes may be a limiting factor with this experiment.

Yes, this is an avenue that we are pursuing but it will require larger blood volumes, as the Reviewer suggests. Our preliminary data suggests that T cells specific against the epitopes used for tetramer development are of low frequency in the circulation of most donors and will likely require a large number of input PBMC in order to yield a population of specific T cells large enough to permit meaningful characterization. However, we agree that it is an approach worth testing. This is discussed in lines 257-261 of the revised manuscript in the context of future studies that could build upon the data reported here.

Typo on lines 151-152 (date/time inserted instead of a reference?)

This has now been corrected.
